# IslandViewer 4: expanded prediction of genomic islands for larger-scale datasets

**DOI:** 10.1093/nar/gkx343

**Published:** 2017-05-02

**Authors:** Claire Bertelli, Matthew R Laird, Kelly P Williams, Simon Fraser University Research Computing Group, Britney Y Lau, Gemma Hoad, Geoffrey L Winsor, Fiona SL Brinkman

**Affiliations:** 1 Department of Molecular Biology and Biochemistry, Simon Fraser University, Burnaby, BC V5A 1S6, Canada; 2 European Molecular Biology Laboratory, European Bioinformatics Institute, Wellcome Genome Campus, Hinxton, Cambridge CB10 1SD, UK; 3 Systems Biology Department, Sandia National Laboratories, Livermore, CA 94551, USA; 4 Big Data Hub, Simon Fraser University, Burnaby, BC V5A 1S6, Canada

## Abstract

IslandViewer (http://www.pathogenomics.sfu.ca/islandviewer/) is a widely-used webserver for the prediction and interactive visualization of genomic islands (GIs, regions of probable horizontal origin) in bacterial and archaeal genomes. GIs disproportionately encode factors that enhance the adaptability and competitiveness of the microbe within a niche, including virulence factors and other medically or environmentally important adaptations. We report here the release of IslandViewer 4, with novel features to accommodate the needs of larger-scale microbial genomics analysis, while expanding GI predictions and improving its flexible visualization interface. A user management web interface as well as an HTTP API for batch analyses are now provided with a secured authentication to facilitate the submission of larger numbers of genomes and the retrieval of results. In addition, IslandViewer's integrated GI predictions from multiple methods have been improved and expanded by integrating the precise Islander method for pre-computed genomes, as well as an updated IslandPath-DIMOB for both pre-computed and user-supplied custom genome analysis. Finally, pre-computed predictions including virulence factors and antimicrobial resistance are now available for 6193 complete bacterial and archaeal strains publicly available in RefSeq. IslandViewer 4 provides key enhancements to facilitate the analysis of GIs and better understand their role in the evolution of successful environmental microbes and pathogens.

## INTRODUCTION

Genomic islands (GIs) are commonly defined as clusters of genes of probable horizontal origin in bacterial or archaeal genomes. They are a major driver of genome evolution and are of particular interest since they often provide adaptive traits that enhance the fitness of bacteria and archaea within a niche (1,2). Indeed, GIs disproportionately encode virulence factors in pathogens (3) and some antimicrobial resistance genes are known to be commonly found in these mobile regions (4–6). They also disproportionately encode novel genes (7), environmentally relevant adaptations like metal resistance (8), and entire metabolic pathways such as for the degradation of monoaromatic hydrocarbons (9). Recently, Ingle *et al*. (10) showed that GIs were the major source of variations between outbreak and non-outbreak strains for atypical enteropathogenic *Escherichia coli*. These and other studies (11,12) further stress the importance of GI predictions for pathogen outbreak analysis.

A large number of methods have been developed to predict and visualize GIs, most of which rely on the recognition of GI features (mobility genes, phage-related genes, direct repeats) and nucleotide composition (13,14). Initially published in 2009 (15), IslandViewer was the first webserver integrating three of the most accurate and complementary GI prediction tools; IslandPath-DIMOB (16) based on nucleotide bias and presence of mobility genes, SIGI-HMM (17) based on codon usage bias with a Hidden Markov Model approach and IslandPick (18) based on a comparative genomics approach. It remains the only webserver to provide integrated GI predictions. GIST, a standalone program with a graphical user interface (19) and its underlying tool EGID (20), is the only other interface that enables the prediction of GIs using a composite set of software. However, due to the selection of integrated tools, EGID features a high recall but a low precision (20) compared to the tools integrated in IslandViewer (18). The IslandViewer webserver also provides precomputed GIs predictions for all microbial genomes available in RefSeq, whereas another resource using similar methodology named PAIDB (21) focuses on pathogenicity and resistance islands only.

The last IslandViewer update, IslandViewer 3, was published in 2015 (22) and provided a novel interactive and flexible visualization on both circular and linear layouts using GenomeD3Plot (23) for 2794 complete bacterial and archaeal genomes with pre-computed predictions. IslandViewer 3 also enabled users to obtain custom predictions through submission of complete chromosomes or draft genomes—the latter being an analysis in high demand but not previously available. To facilitate the identification of pathogenicity islands of special interest in clinical microbiology, pre-computed genomes displayed additional annotations of 28 911 antimicrobial resistance (AMR) genes identified using the Resistance Gene Identifier (RGI) from the Comprehensive Antibiotic Resistance Database (CARD) (24), 39 441 probable orthologs of virulence factors (VF) from the Virulence Factor Database (VFDB) (25), PATRIC (26) and Victor's virulence factors (http://www.phidias.us/victors/) as well as 18 919 pathogen-associated genes (3,22).

To meet the current needs in public health and fundamental microbiology for large-scale analysis of microbial genomes, the IslandViewer webserver now provides for the first time a programmatic interface and a user management system for more flexible automated submission of genomes and retrieval of results. IslandViewer has been partially re-written to provide much faster and expanded GI predictions displayed in a streamlined interactive layout to produce publication grade figures while retaining its flexibility. These improvements, including the integration of the latest version of IslandPath-DIMOB, and newly added predictions from Islander (27) based on the frequent use of tRNA and tmRNA genes as integration sites, are released as IslandViewer 4, the most comprehensive and flexible GI analysis webserver currently available.

## EXPANDED GENOMIC ISLAND AND GENE CONTENT PREDICTIONS

Pre-computed analysis of publicly available genomes is a valuable resource provided by IslandViewer which has previously integrated three GI prediction methods as well as annotations of VFs, pathogen-associated genes, and AMR genes. To reflect major changes in publicly available microbial genomes in the present IslandViewer 4 release, GI predictions have been newly computed for the 6193 complete bacterial and archaeal strains (encompassing 6586 chromosomes) currently publicly accessible in RefSeq (ftp.ncbi.nlm.nih.gov/genomes/refseq/bacteria) as retrieved by MicrobeDBv2 (28) on 9 February 2017. In particular, IslandPick predictions have been entirely recomputed for all chromosomes to benefit from the larger number of genomes available for comparison. Moreover, the virulence factor analysis has been updated to use the new non-redundant protein accession numbers issued from the NCBI genome reannotation initiative of RefSeq. AMR genes have been newly predicted using RGI with the latest CARD database (6 February 2017) (24), leading to the identification of 10,826 perfect matches to the curated reference sequences of CARD and 166 193 genes with a strict cutoff identifying novel variants of known AMR genes. Note that pathogen-associated genes are contextual, based on a previous analysis (22), and dependent on the genomes compared to, so researchers are encouraged to perform additional analyses to confirm their potential interest. Old accession numbers were also conserved to enable curated annotation transfer for users submitting older genome files. All pre-computed analyses will continue to be updated semiannually with newly released genomes. During these regular updates, predictions are computed only for genomes newly available in RefSeq and genomes with a different version of the accession number (indicating changes in the nucleotide sequence) to keep consistent predictions in the pre-computed database. Changes in annotation, especially due to the recent NCBI reannotation initiative for all genomes in RefSeq, that might impact IslandPath-DIMOB predictions as well as the availability of additional genomes for IslandPick comparative genomics approach are not considered. Note that IslandViewer 3 and its pre-computed results will remain available with an alternative url (http://www.pathogenomics.sfu.ca/islandviewer3).

**Figure 1.**
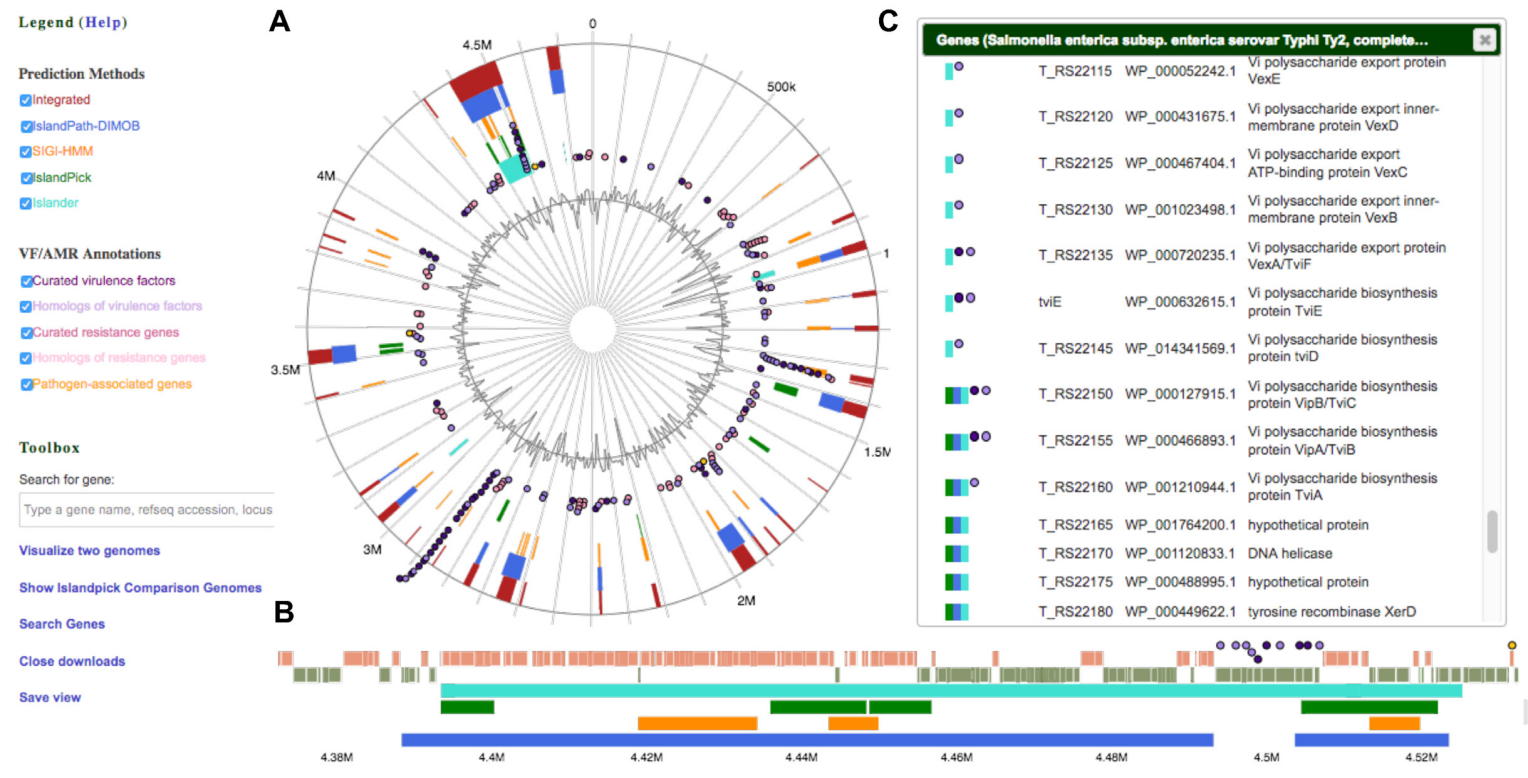
Example IslandViewer 4 results. Predictions of genomic islands in the *Salmonella enterica* str. Ty2 genome (accession number NC_004631.1). Circular (**A**) and linear (**B**) visualization of predicted genomic islands are shown, with blocks colored according to the prediction method; IslandPick (green), IslandPath-DIMOB (blue), SIGI-HMM (orange), Islander (turquoise) as well as the integrated results (dark red). Virulence genes (purple for curated, light purple for homologs), antimicrobial resistance genes (pink for curated, light pink for homologs) and pathogen-associated genes (yellow) are also visible as circular glyphs. Tickboxes in the legend enable users to turn on/off individual items in the circular view. Double clicking on any region zooms into the region in panel (B) and opens a vertical view of the genes and their description (**C**) within the region selected in the circular view. Circular or horizontal views can be exported to obtain publication-grade customized figures.

To further improve the quality of the proposed GI predictions, IslandViewer 4 now includes predictions from the previously published Islander database (27), a fourth highly precise method that specifically predicts GIs integrated into tRNAs and tmRNAs with a precise definition of boundaries. In total, 3557 predicted GIs can be interactively browsed using the web interface (Figure 1) in 1264 bacterial and archaeal strains, among the 2168 genomes analyzed. The assessment of Islander predictions accuracy using a reference subdataset from Langille *et al*. (18) shows a low recall (21%) but the highest precision (91%) among prediction tools integrated in IslandViewer 4. Recall is low because Islander insists that the island both contain an integrase gene of the tyrosine recombinase family and be integrated into a tRNA/tmRNA gene, while the reference dataset does not. Precision is high because Islander finds the RNA gene fragment split off during integration that marks the distal end of the island. Islander predictions provide a second method, in addition to IslandPick (18), to define more accurately GI boundaries and complement well the two other predictive methods, namely SIGI-HMM (29) and IslandPath-DIMOB (7), already integrated in IslandViewer that predict GI regions with less boundary accuracy but overall higher recall.

As the number of genomes increases, refinements in GI predictions need to be updated to reflect current GI knowledge based on comparative genomics. IslandViewer 4 integrates an improved version of IslandPath-DIMOB (https://github.com/brinkmanlab/islandpath) with updated Pfam profiles for identification of mobility genes and more stringent significance cutoffs to avoid false positives, a more sensitive identification of regions with biased dinucleotide score, as well as the merging of close predictions to correct the previously observed fragmented GI predictions. Overall, IslandPath-DIMOB v1.0.0 features a 11.7% increase in recall and a 5.3% increase in precision compared to the currently available version in IslandViewer when assessed using the same dataset (18). Both pre-computed and user-submitted custom genome analysis benefit from the new predictions by IslandPath-DIMOB v1.0.0.

IslandViewer 4 has retained the flexible interactive interface for visualization of GIs and their gene content using GenomeD3Plot (23) (formerly called IslandPlot) that was a major improvement reported with the previous release. A new approach was implemented to speed up the loading of GI prediction results in the interactive visualization. Furthermore, the layout and colours of the circular view have been streamlined (Figure 1) to directly produce a publication-grade figure that can be downloaded to vector or image format. The side-by-side visualization of two genomes has been improved to provide a gene pop-up window for each genome, thus facilitating the analysis of the gene content.

## WEB AND PROGRAMMATIC USER INTERFACE FOR LARGER SCALE ANALYSES

During the past decade, the number of researchers using IslandViewer and the number of genomes submitted per user (based on statistics of users providing an email address) has steadily increased. Previously, users had to submit custom genomes individually through the web interface and had to bookmark the link to access the results or to provide an e-mail address to be notified of completion of GI predictions. While this method allowed easy access to GI predictions for individual genomes, it did not enable large-scale analysis of closely-related bacterial genomes, as is currently needed to investigate pathogen outbreaks and more widely to study microbial genome evolution (1,30).

Therefore, an HTTP API is now provided to facilitate the submission of larger numbers of genomes and the retrieval of GI prediction results without the tedious process of clicking through multiple links and manually typing optional fields through the web interface. Using the programmatic interface, users can query the availability of reference genomes in IslandViewer 4 database prior to submitting draft genomes that require a reference genome for contig reordering. Similarly, users can query and select reference genomes to run IslandPick with custom distance parameters. The HTTP API can be used as an anonymous user with an empty security token or as an authenticated user, which enables further functionality such as retrieving information about previously submitted genomes (Supplementary Figure S1). In addition, a management web interface has been developed to allow users to securely view genomes they had previously submitted with a link to directly access the dynamic visualization of results (Supplementary Figure S1). Results of custom genome analyses remain available for at least 3 months. To use the management web interface and the HTTP API in authenticated mode, users must first login using Github, Google or Twitter credentials via a standard OAuth authorization framework. After a user's first login, a user-specific secured token is generated that enables use of the HTTP API in authenticated mode. In parallel, the security to access GI predictions and download results has been improved to ensure high standards of data privacy for user-submitted custom genomes. Overall, the use of a login and the provision of other information such as email addresses remains optional.

## IMPROVED GENOMIC DISTANCE COMPUTATION USING MASH FOR FASTER TIME-TO-RESULTS

The large number of complete reference genomes available in IslandViewer has significantly increased the time (up to several hours) to compute pairwise genomic distance calculations using CVTree (31) that enabled the automatic or user-custom selection of genomes for IslandPick comparative genomics analysis. Therefore, to ensure our ability to handle the anticipated larger number of predictions needed thanks to the new batch submission system, IslandViewer 4 now integrates MASH (32) – a recently published tool for efficient measure of genome similarity – as a replacement of CVTree (31). MASH reduces the complexity of genome sequence by representing a single genome with a number (by default 1000) of sequence k-mers (default 21-mers) in a so-called sketch. CVTree distances <0.42 – a cutoff implemented in IslandViewer 3 – and MASH distance yield a Pearson correlation coefficient of 0.97 when genomes are sketched to 10 000 *k*-mers of default length. A linear regression enabled us to directly translate the previously used CVTree distance cutoffs of 0.1–0.42 into corresponding MASH distance cutoffs of 0–0.21 to pick reference genomes for IslandPick predictions (Supplementary Figure S2). CVTree and MASH distance measures are highly correlated, enabling optimal preservation of the ability to compare a genome with other strains as previously offered in IslandViewer. Previously, CVTree took hours to calculate the distance of a custom genome with all available reference microbial genomes whereas using MASH reduces this analysis time to 1.2 s on average. This is a significant decrease in time-to-results – from hours to minutes – to obtain GI predictions from the entire IslandViewer pipeline.

## OTHER BACKEND UPGRADES

IslandViewer 4 comes with a new database backend using MariaDB, a community-based fork of MySQL with support for the Percona XtraDB storage engine. XtraDB provides performance enhancements over the previous InnoDB storage engine that combine with the existing robust queuing system to improve scalability and should avoid intermittent problems. It will better handle the rapidly increasing number of reference genomes available as pre-computed results and will ensure a faster retrieval of results for users from the web and the HTTP API interface.

## DISCUSSION AND CONCLUDING REMARKS

The prediction of GIs remains an essential analysis for newly sequenced bacterial and archaeal genomes, and IslandViewer has proven over time to be a widely appreciated resource to predict and explore GIs interactively. IslandViewer 4 now integrates better tools to predict GIs (Table 1) that includes a higher recall with IslandPath-DIMOB v1.0.0 and a better definition of some GI boundaries thanks to the integration of Islander predictions (27). However, for users wishing to pursue a previously started analysis, IslandViewer 3 will remain available with an alternative URL (http://www.pathogenomics.sfu.ca/islandviewer3). IslandViewer 4 also fills a gap by enabling rapid large-scale analysis of genomes with multiple highly accurate GI prediction tools through the HTTP API. Previously, only EGID enabled batch prediction of GIs using a combination of five tools, although with a lower precision (0.63) than the methods incorporated in IslandViewer which all reach over 0.85 in precision (20). Furthermore, IslandViewer is the only currently active web service enabling GI analysis for draft (incomplete, not closed) as well as complete genomes. Some components of IslandViewer pipeline, including SIGI-HMM, IslandPath-DIMOB and the virulence factor modules, require an annotated genome with predicted coding sequences. Thus, IslandViewer only accepts gbk and embl file formats as input.

**Table 1.** Comparison of IslandViewer 3 and IslandViewer 4 webservers: methods and available resources

	IslandViewer 3	IslandViewer 4
**Pre-computed predictions at the time of publication**		
*Number of genomes available*	2794 bacteria and archaea	6193 bacteria and archaea
*GI prediction methods*	IslandPath-DIMOB v0.2	IslandPath-DIMOB v1.0.0
	SIGI-HMM	SIGI-HMM
	IslandPick	IslandPick
		Islander
*Antimicrobial resistance genes*	RGI from CARD	RGI from CARD
	28 911 genes identified	177 019 genes identified
*Virulence factors*	VFDB, PATRIC and Victor's virulence factors	VFDB, PATRIC and Victor's virulence factors
	39 441 genes identified	189 161 genes identified
*Pathogen-associated genes*	18 919 genes identified	25 981 genes identified
		
**Custom predictions**		
*Input file*	GenBank or EMBL	GenBank or EMBL
*GI prediction methods*	IslandPath-DIMOB v0.2	IslandPath-DIMOB v1.0.0
	SIGI-HMM	SIGI-HMM
	IslandPick	IslandPick
*Virulence factors*	Transfer of curated annotation based on protein accession number (genome specific)	Transfer of curated annotation based on non-redundant protein accession number (sequence specific)
		
**Genomic distance calculation**	CVTree	MASH
**Time-to-results for new custom genome analysis^+^**	Hours	Minutes
**API for batch submission**	None	Anonymous or authenticated
User management interface*	None	Authenticated
**Interactive visualization**	Circular and linear plots	Circular and linear plots

^+^Results for custom genomes previously submitted are immediately available.

*Custom genome analysis results remain available for 3 months.

The incorporation of cutting-edge developments in comparative genomics, by incorporating MASH (32) to estimate between-genome distance, ensures much faster processing of a user's custom genome than in the previous release – changing run time from hours to minutes. Collectively, these improvements have made it possible to integrate IslandViewer 4 in the pipeline of the Integrated Rapid Infectious Disease Analysis platform (www.IRIDA.ca) used by public health agencies for pathogen outbreak analysis. More development is required in the future to facilitate viewing of GI predictions in many genomes at once, in a comparative format and we have initiated development of a new tool to meet this need. Meanwhile, building on the flexible visualization tools developed in the previous release, IslandViewer 4 remains a user-friendly interface for researchers to evaluate the presence of VFs, AMR genes and pathogen-associated genes within and outside predicted genomic islands as highlighted in Figure 1. Overall, the improvements proposed by IslandViewer 4 will enhance the study of GIs and their role in the evolution of a wide range of bacterial and archaeal species of interest and investigations into the acquisition of antimicrobial resistance and other medically important adaptations.

## Supplementary Material

Supplementary Data
