# Supplementary material for: IslandViewer 4: expanded prediction of genomic islands for larger-scale datasets
Source: Nucleic Acids Res. 2017 May 2;45(Web Server issue):W30–5. doi: 10.1093/nar/gkx343 (PMC5570257; doi:10.1093/nar/gkx343)
Supplement: Supplementary Data [file gkx343_Supp.pdf]

SUPPLEMENTARY FIGURES

A Logging in

1. Click on the LOGIN menu and select one of the following: Github, Google or Twitter

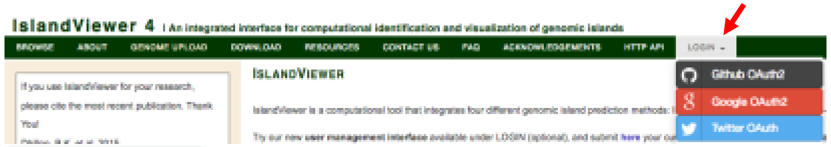

2. Enter your credentials on the selected website (example github) and sign in.
You will be redirected to IslandViewer

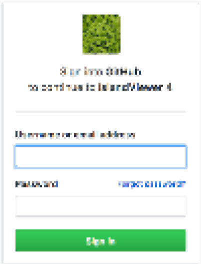

3. IslandViewer interface greets you with your username and now shows an additional JOBS and LOGOUT menus.

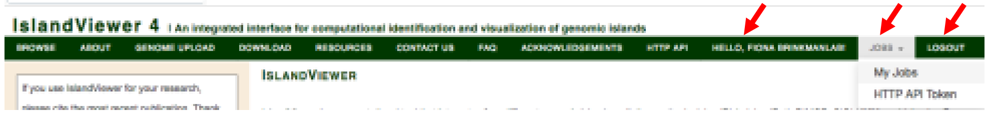

B My Jobs - Web management interface

B.4. Upload genomes, using the usual Genome Upload interface

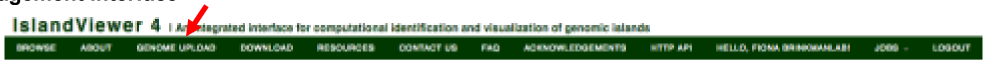

B.5. Click on the JOBS menu and select "My Jobs"
A list of genomes previously submitted when authenticated is available with links to results (in blue) under Token id.

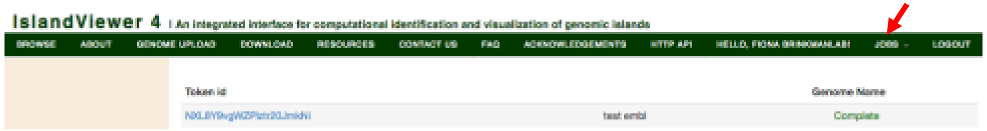

C HTTP API – Batch submission as an authenticated user

C.4. Click on the JOBS menu and select "HTTP API Token".

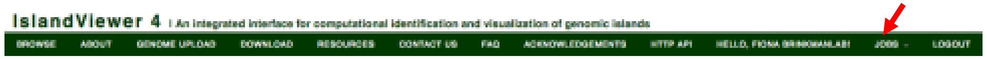

C.5. To use the API as an authenticated user, place your unique user token (in the red rectangle) in your scripts or cURL command, as described in the HTTP API documentation.

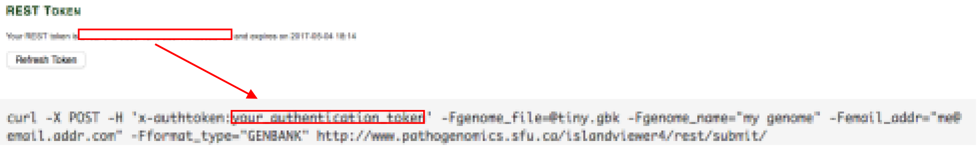

C.6. If your token has expired, click on "Refresh Token" to obtain a new token.

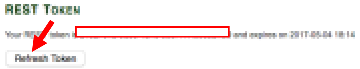

Supplementary Figure 1. Using the web management interface and the HTTP API in authenticated mode.

(A) Users first need to authenticate into the web interface using the login menu. Then, users can submit genomes using the usual genome upload interface (B.4) and access a list of previously submitted genomes in the web management interface (B.5). Users can also see their unique user token (C.4) to use the HTTP API as an authenticated user (C.5). A new user token can be obtained at any time (C.6), if the previous token has expired, or if the privacy of the token was compromised (for example if the user has publicly displayed his user token on a public platform such as github). Genomes submitted using the HTTP API as an authenticated user (C) will become visible in the web management interface (B).

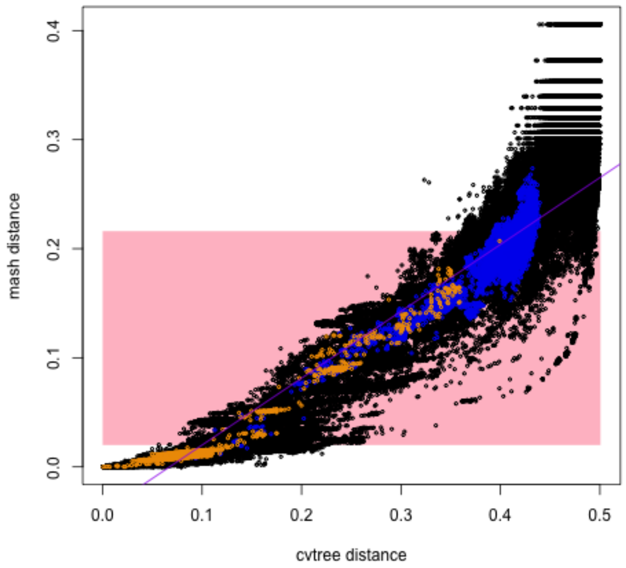

Supplementary Figure 2. Scatterplot of CVTree and MASH distance measures for reference genomes.

CVTree distance below 0.42 (a cutoff previously used in IslandViewer 3) and MASH distance measures show a Pearson correlation coefficient of 0.97. The linear regression between both distance values is shown in purple. The pink area highlights genomes that are available for GI prediction by comparative genomics with default IslandPick parameters. As an illustration, distances between genomes from the *Pseudomonas* genus belonging to the same species (orange) and to different species (blue) are highlighted.
